# Effects of TiC, Si, and Al on Combustion Synthesis of Ti_3_SiC_2_/TiC/Ti_5_Si_3_ Composites

**DOI:** 10.3390/ma16186142

**Published:** 2023-09-09

**Authors:** Chun-Liang Yeh, Kuan-Ling Lai

**Affiliations:** Department of Aerospace and Systems Engineering, Feng Chia University, Taichung 40724, Taiwan

**Keywords:** Ti_3_SiC_2_, TiC, combustion synthesis, weight percentage, X-ray diffraction

## Abstract

The fabrication of Ti_3_SiC_2_ from TiC-containing reactant compacts was investigated by combustion synthesis in the mode of self-propagating high-temperature synthesis (SHS). The initial sample composition was formulated based on (3 − x)Ti + ySi + (2 − x)C + xTiC + zAl, with stoichiometric parameters of x from 0 to 0.7, y = 1.0 and 1.2, and z = 0 and 0.1. For all samples studied, combustion was sufficiently exothermic to sustain the reaction in the SHS manner. Due to the dilution effect of TiC, combustion wave velocity and reaction temperature substantially decreased with TiC content. When compared with the TiC-free sample, the TiC-containing sample facilitated the formation of Ti_3_SiC_2_ and the TiC content of x = 0.5 produced the highest yield. Excess Si (y = 1.2) to compensate for the evaporation loss of Si during combustion and the addition of Al (z = 0.1) to promote the phase conversion were effective in improving the evolution of Ti_3_SiC_2_. All final products were composed of Ti_3_SiC_2_, TiC, and Ti_5_Si_3_. For the TiC-containing samples of x = 0.5, the weight fraction of Ti_3_SiC_2_ increased from 67 wt.% in the sample without extra Si and Al to 72 wt.% in the Si-rich sample of y = 1.2 and further up to 85 wt.% in the Si-rich/Al-added sample of y = 1.2 and z = 0.1. As-synthesized Ti_3_SiC_2_ grains were in a thin plate-like shape with a thickness of 0.5–1.0 μm and length of about 10 μm. Ti_3_SiC_2_ platelets were closely stacked into a layered structure.

## 1. Introduction

MAX phases belong to a family of hexagonal-structure layered ceramics with stoichiometry of M*_n_*_+1_AX*_n_* (usually *n* = 1, 2, or 3), where M is an early transition metal (Ti, Zr, Nb, Ta, Cr, etc.), A is an element of the IIIA or IVA group (Al, Si, Sn, Ge, etc.), and X is C or N [1]. Typical MAX phases such as Ti_2_AlC, Cr_2_AlC, Ti_3_SiC_2_, Ti_3_AlC_2_, Nb_4_AlC_3_, and Ti_4_AlN_3_ as well as their associated solid solutions have been broadly investigated [1,2,3,4,5]. Properties of the MAX phases combine merits of both metals and ceramics. Like metals, they are excellent thermal and electrical conductors, readily machinable, and highly resistant to thermal shock. Like ceramics, they possess high stiffness, low density, low thermal expansion coefficients, and good corrosion and oxidation resistances [6,7]. In addition, higher-order MAX phases with *n* > 3 such as Ta_5_AlC_4_, Ta_6_AlC_5_, Ti_6_AlC_5_, and Ti_7_SnC_6_ have been discovered [8,9,10].

Potential applications of MAX ceramics have been considered, including structural materials for high-temperature applications, protective coatings and bond-coat layers in thermal barrier systems, accident-tolerant fuel cladding in nuclear power plants, solar receivers in concentrated solar power systems, electrical contacts, catalysts, heat exchangers, corrosion-resistant materials, and joining materials [1,11]. Specifically, Ti_2_AlC, Ti_3_AlC_2_, and Cr_2_AlC are potential candidates to replace Ni/Co superalloys in the hottest part of a gas turbine engine to enable operation at higher temperatures [12]. Ti_3_SiC_2_, Ti_2_AlC, Ti_3_AlC_2_, and Cr_2_AlC have attracted considerable attention as potential accident-tolerant fuel claddings in light-water nuclear reactors [13]. Ti_3_SiC_2_ and Ti_2_AlC have been proposed as ohmic contacts [14]. As catalysts, Ti_3_SiC_2_, Ti_2_AlC, and Ti_3_AlC_2_ show excellent chemoselectivity for the hydrogenation of organic compounds [15].

Ti_3_SiC_2_ is one of the most studied M_3_AX_2_ compounds and has been produced by many powder-sintering processes such as hot pressing (HP) [16], hot isostatic pressing (HIP) [17], mechanical alloying (MA) [18,19], spark plasma sintering (SPS) [20,21,22,23], and self-propagating high-temperature synthesis (SHS) [24,25,26,27]. A variety of powder combinations have been adopted as the initial reactants, including Ti/Si/C, Ti/SiC/C, Ti/Si/TiC, Ti/TiSi_2_/TiC, Ti/SiC/TiC, TiH_2_/SiC/C, etc. [16,17,18,19,20,21,22,23,24,25,26,27]. Sintering fabrication routes often produce secondary phases (e.g., TiC_x_, TiSi_2_, Ti_5_Si_3_, SiC, and Ti_5_Si_3_C_x_) along with Ti_3_SiC_2_. Although TiC and Ti_5_Si_3_ are considered to be suitable reinforcing phases for Ti_3_SiC_2_ [28,29], several approaches have been applied to enhance the production of Ti_3_SiC_2_.

For the improvement of Ti_3_SiC_2_ formation, most of the studies have focused on different reactant mixtures, off-stoichiometric compositions, and Al additions. For example, Radhakrishnan et al. [30] synthesized Ti_3_SiC_2_ from element powders with a Si-excess composition of Ti:Si:C = 3:1.2:2 by reactive sintering at 1800 °C for 10 h. Liu et al. [31] employed TiH_2_, Si, and graphite powders under a Si-rich stoichiometry of Ti:Si:C = 3:1.2:2 to prepare porous Ti_3_SiC_2_ through a multi-stage sintering process, within which TiH_2_ decomposed to form Ti at 600 °C, followed by the formation of TiC and Ti_5_Si_3_ at about 1100 °C, and the production of Ti_3_SiC_2_ at 1350 °C. Li et al. [28] indicated that an increase in Si increased the fraction of Ti_3_SiC_2_ in a Ti_3_SiC_2_–Ti_5_Si_3_–TiC composite fabricated by reactive plasma spraying. It was believed that excess Si compensated for the evaporation loss of Si at high temperatures and, therefore, led to a higher yield of Ti_3_SiC_2_. With the aid of Al as a reaction promoter in the SPS process, Zhang et al. [32] prepared Ti_3_SiC_2_ from an element powder mixture of 3Ti/Si/2C/*x*Al (*x* = 0.1–0.3) at 1280 °C, and Sun et al. [33] obtained Ti_3_SiC_2_ from the reactant mixtures of 3Ti/SiC/C/*x*Al (*x* = 0.15 and 0.2) at 1200 °C. The effect of Al additions on enhancing the formation of Ti_3_SiC_2_ was also confirmed by pressureless sintering of reactant mixtures composed of 4Ti/TiC/2SiC/0.2Al at 1380 °C for 1 h [34] and 3Ti/1.5Si/1.9C/0.5Al at 1400 °C for 2 h [35]. Moreover, Gubarevich et al. [36] utilized an Al-containing element powder mixture of 3Ti/Si/2C/0.1Al to produce Ti_3_SiC_2_ by combustion synthesis. It has been proposed that Al could suppress the evaporation of Si at high temperatures and form a liquid phase in the Ti-Si-C-Al quaternary system, which could facilitate the diffusion of Ti and Si atoms and promote the interactions between TiC and Ti_5_Si_3_ or Ti-Si melt [34,35,36].

However, the influence of excess Si and Al on the fabrication of Ti_3_SiC_2_ by combustion synthesis with raw materials containing TiC has been little studied. As a first attempt, this study aimed to investigate the effects TiC, Si, and Al on the formation of Ti_3_SiC_2_ by solid-phase combustion synthesis in the SHS mode. When compared with other processing methods, the SHS technique takes advantage of highly exothermic reactions to produce MAX phases and has benefits of energy effectiveness, time-saving, simplicity, low cost, scalability, and high-purity products [37,38,39]. In this study, starting mixtures comprising different amounts of TiC were prepared with an exact Ti:Si:C = 3:1:2 stoichiometry and Si-rich and Al-added compositions. The effects of TiC content and excess Si and Al were studied on the combustion wave velocity and reaction temperature as well as on the enhancement of Ti_3_SiC_2_ formation.

## 2. Materials and Methods

The starting materials adopted by this study included Ti (Alfa Aesar, Ward Hill, MA, USA, <45 μm, and 99.8%), Si (Strem Chemicals, Newburyport, MA, USA, <45 μm, and 99.5%), carbon black (Showa Chemical Co., Tokyo, Japan), TiC (Aldrich Chemical, Burlington, MA, USA, <45 μm, and 99%), and Al (Showa Chemical Co., Tokyo, Japan, <45 μm, and 99%). The reactant mixture was formulated based on the following equation:

(1) $$\left(3-x\right)Ti+ySi+(2-x)C+xTiC+zAl\to Ti_{3}SiC_{2}$$

where the parameters x, y, and z signify the numbers of moles of TiC, Si, and Al in the reactant mixture, respectively. In this study, the content of TiC was studied with x = 0, 0.2, 0.3, 0.4, 0.5, 0.6, and 0.7. The sample of x = 0 was TiC-free and composed only of elemental Ti, Si, C, and Al powders. Two mole numbers of y = 1.0 and 1.2 were examined for Si; y = 1.0 denoted a sample without extra Si and y = 1.2 represented a sample with Si in excess of 20 mol.%. The value of z defined a sample without (z = 0) or with Al (z = 0.1) additions. The amount of Al included in the reactant mixture was very small. In summary, three sample compositions with different contents of TiC were involved; namely, the exact stoichiometry of Ti:Si:C = 3:1:2, a Si-rich configuration of Ti:Si:C = 3:1.2:2, and a Si-rich/Al-added composition of Ti:Si:C:Al = 3:1.2:2:0.1. Reactant powders were dry-mixed in a ball mill and uniaxially compressed into cylindrical samples with a diameter of 7 mm, a height of 12 mm, and a relative density of 50%.

The combustion synthesis experiment was conducted in a windowed stainless-steel chamber under an Ar environment at a pressure of 0.25 MPa. A tungsten coil with an electric voltage of 60 V and a current of 1.5 A was mounted at 1 mm above the sample top surface and was utilized to ignite the sample by thermal radiation. The combustion wave propagation velocity (V_f_) was determined from the flame-front trajectory, based on the time sequence of recorded pictures. The exposure time of each recorded image was set at 0.1 ms. To facilitate the accurate measurement of instantaneous locations of the combustion front, a beam splitter with a mirror characteristic of 75% transmission and 25% reflection was used to optically superimpose a scale onto the image of the sample. The combustion temperature was measured by a bare-wire thermocouple (Pt/Pt-13%Rh) with a bead size of 125 μm. Details of the experimental setup and approach have been reported elsewhere [40]. The phase composition and microstructure of the final product were analyzed using an X-ray diffractometer (XRD, Bruker D2 Phaser, Karlsruhe, Germany) with CuK_α_ radiation and a scanning electron microscope (SEM, Hitachi, S3000H, Tokyo, Japan), respectively. Based on the XRD peak intensity, the weight fractions of Ti_3_SiC_2_, Ti_5_Si_3_, and TiC in the product were calculated by the following equations [25,41]:

$$W_{TSC}=\frac{I_{TSC}}{I_{TSC}+4.159I_{TS}+0.818I_{TC}}$$

$$W_{TS}=\frac{I_{TS}}{I_{TS}+0.24I_{TSC}+0.197I_{TC}}$$

$$W_{TC}=\frac{I_{TC}}{I_{TC}+1.222I_{TSC}+5.084I_{TS}}$$

where *W_TSC_*, *W_TS_*, and *W_TC_* are the weight percentages of Ti_3_SiC_2_, Ti_5_Si_3_, and TiC, respectively. *I_TSC_*, *I_TS_*, and *I_TC_* are the integrated intensities of diffraction peaks associated with Ti_3_SiC_2_ (104) at 2θ = 39.548°, Ti_5_Si_3_ (102) at 2θ = 37.565°, and TiC (111) at 2θ = 35.918°, respectively [25,41]. The equations derived by Zou et al. [41] were based on the theory of X-ray diffraction, in that the diffraction intensity of *i*th phase is a function of the mass fraction of the ith phase in the sample and the overall absorption coefficient of the sample. The equations were specifically applied for a quantitative evaluation of the contents of three coexisting phases; namely, Ti_3_SiC_2_, Ti_5_Si_3_, and TiC. The constants of the equations were experimentally determined from the XRD patterns of the powder mixture samples, which were blended from pure Ti_3_SiC_2_, Ti_5_Si_3_, and TiC at weight ratios of 2:4:4, 3:2:5, 4:3:3, and 5:4:1. Representative reflections of Ti_3_SiC_2_ (104), Ti_5_Si_3_ (102), and TiC (111) were chosen because they did not overlap with other peaks in the XRD patterns [41].

## 3. Results and Discussion

### 3.1. Combustion Wave Kinetics

**Figure 1:**
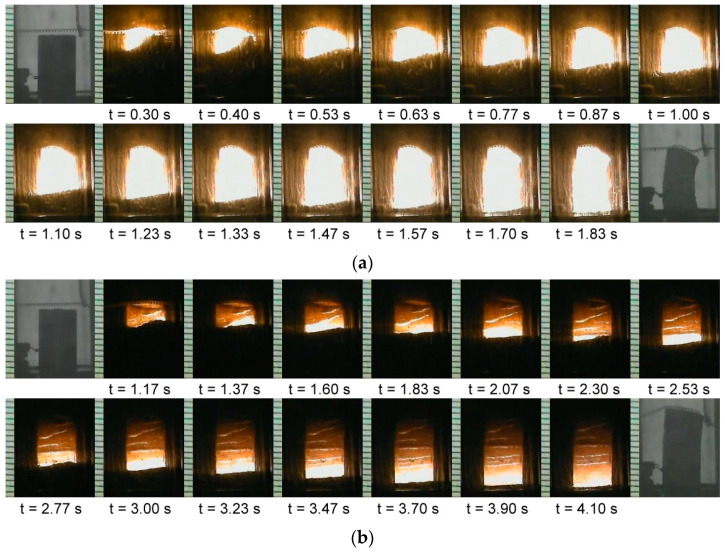
Time sequences of recorded SHS images illustrating the propagation of self-sustaining combustion waves of Al-free samples with (**a**) TiC of x = 0.2 and Si of y = 1 and (**b**) TiC of x = 0.7 and Si of y = 1. The increase in TiC reduced the combustion intensity and decelerated the combustion wave.

Experimental observations of this study indicated that combustion wave behavior of the sample compact apparently varied with the TiC content in the starting mixture. For the 3Ti-Si-2C stoichiometric sample with TiC of x = 0.2, Figure 1a shows that upon ignition, a distinct combustion front formed and propagated downward in a self-sustaining manner. As revealed in Figure 1a, the combustion wave traversed the entire sample in about 1.83 s and a slightly bent deformation of the burned sample was observed, perhaps due to a liquid phase being formed during the SHS process. With an increase in TiC content to x = 0.7, as presented in Figure 1b, the combustion wave exhibited a spinning propagation mode. According to Ivleva and Merzhanov [42], this is because the heat flux liberated from self-sustaining combustion is no longer sufficient to maintain the steady propagation of a planar front. There are both thermodynamic and kinetic reasons that provide the basis for departure from a steady condition [43]. Thermodynamic considerations arise from the degree of exothermicity of the reaction. Kinetic reasons are largely attributed to insufficient reactivity due to the presence of diffusion barriers. The spinning combustion wave observed in Figure 1b could have been primarily caused by the dilution effect of TiC on reaction exothermicity, which resulted in a longer combustion spreading time of about 4.10 s. It should be noted that the addition of extra Si (y = 1.2) and Al (z = 0.1) had almost no effect on the combustion behavior.

**Figure 2:**
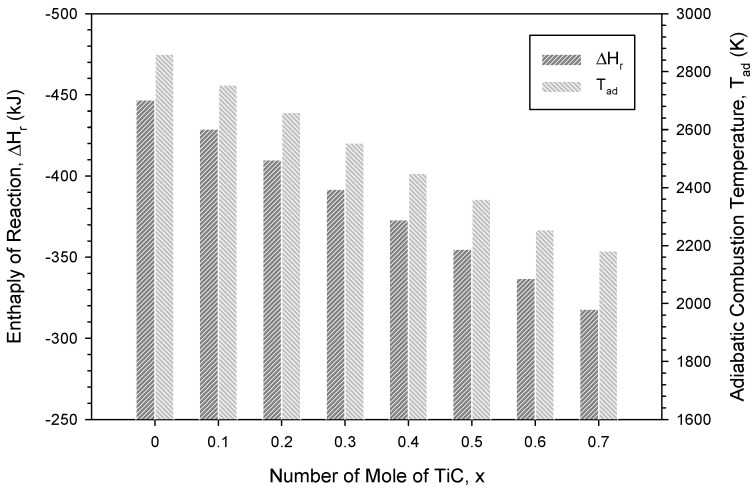
Enthalpies of reaction (ΔH_r_) and adiabatic combustion temperatures (T_ad_) of Equation (1) as a function of number of moles of TiC.

The enthalpy of reaction (ΔH_r_) and adiabatic combustion temperature (T_ad_) of Equation (1) with y = 1 and z = 0 were calculated as a function of the TiC content (i.e., x = 0–0.7) from the energy balance equation with thermochemical data [44,45] and plotted in Figure 2. For the TiC-free sample (x = 0), Figure 2 shows that the reaction had the largest ΔH_r_ of −448 kJ and the highest T_ad_ of about 2860 K. The increase in TiC content from x = 0.1 to 0.7 led to a decrease in ΔH_r_ from −430 to −320 kJ and a considerable decline in T_ad_, from 2754 K to 2182 K. The calculated results of Figure 2 provide an explanation for the observed combustion behavior.

**Figure 3:**
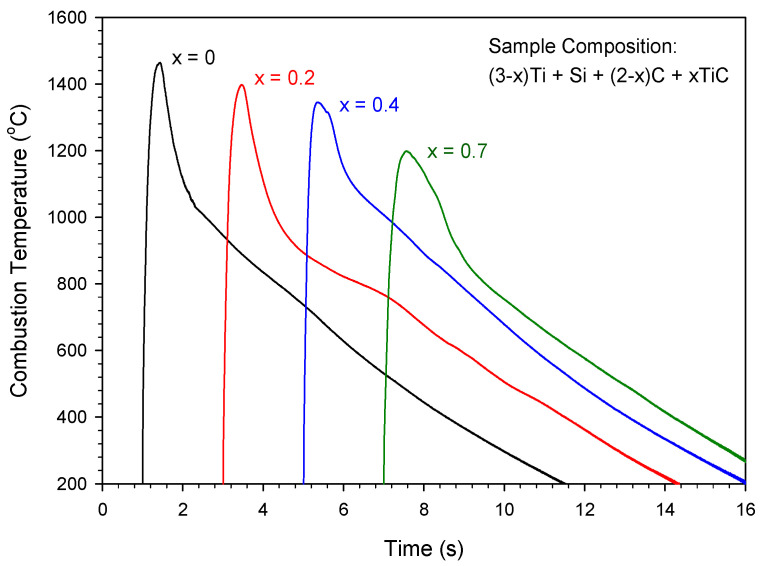
Effect of TiC content on the combustion temperature of reactant compacts for synthesis of Ti_3_SiC_2_.

Figure 3 plots the temperature profiles measured from the samples without extra Si and Al, but containing different amounts of TiC. A temperature profile detected from a TiC-free element powder compact (x = 0) was included for comparison. All profiles exhibited a steep temperature rise followed by a rapid descent, which is typical of the SHS reaction that features a fast combustion wave and a thin reaction zone. The peak value was considered as the combustion front temperature (*T*_c_). Due to the dilution effect of TiC on combustion, Figure 3 shows a decrease in peak combustion temperature from 1464 °C for the TiC-free sample to about 1200 °C for the TiC-containing sample of x = 0.7. The descending trend was consistent with the calculated adiabatic temperature. On the other hand, the addition of extra Si and a small amount of Al essentially had little influence on the combustion temperature. Experimental measurements indicated that the temperature variation induced by adding extra Si and Al was within ±20 °C of the value detected from the 3Ti-Si-2C stoichiometric sample. 

It was found that the reaction front temperatures of the samples with x = 0, 0.2, and 0.4 were above the lowest eutectic point (1330 °C) of the Ti–Si mixture. This justified the reaction mechanism proposed by Gauthier et al. [46], which suggested that Ti_3_SiC_2_ is formed through the interaction of a Ti–Si liquid phase with solid TiC_x_. However, the peak combustion temperatures of the TiC-added samples of x = 0.5, 0.6, and 0.7 varied between 1200 and 1300 °C; therefore, the formation of Ti_3_SiC_2_ could have been dominated by solid-phase reactions. For a mechanism involving no liquid phases [47,48], Ti_3_SiC_2_ is proposed to form through two intermediates, TiC_x_ and Ti_5_Si_3_C_x_, reacting with free silicon and carbon, respectively. This implies that an increase in TiC content in the starting composition could lead to a transition of the governing reaction mechanism from a liquid–solid scenario to a solid-state mode.

It was proposed that the reaction mechanism of Ti_3_SiC_2_ formation in the Ti-Si-C system could be initiated by the reaction of Ti with C as Equation (2), followed by the interaction between Ti and Si as Equation (3). These two reactions are highly exothermic and produce TiC and Ti_5_Si_3_ as the precursors. Finally, the formation of Ti_3_SiC_2_ is via a reaction involving TiC, Ti_5_Si_3_, and Si, as shown in Equation (4).

(2) Ti+C→TiC

(3) $$5Ti+3Si\to Ti_{5}Si_{3}$$

(4) $$2TiC+0.2Ti_{5}Si_{3}+0.4Si\to Ti_{3}SiC_{2}$$

**Figure 4:**
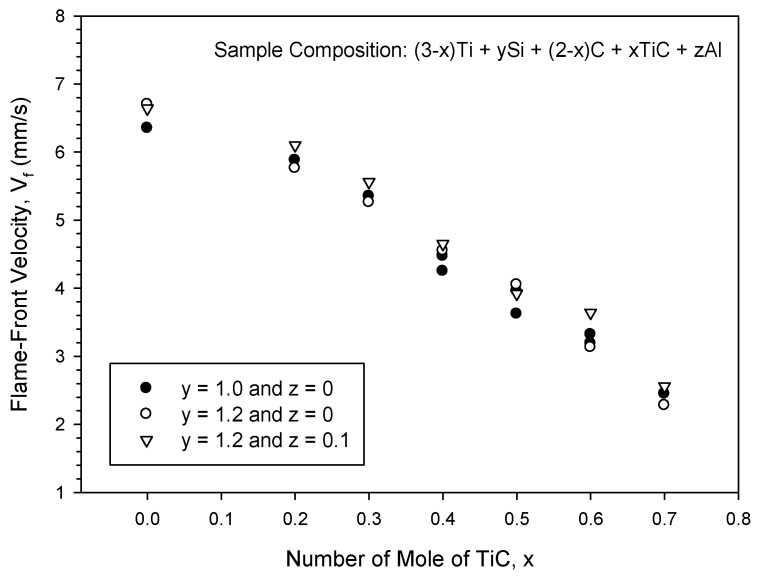
Effects of TiC, Si, and Al on the combustion front velocity of reactant compacts for synthesis of Ti_3_SiC_2_. Combustion velocity was strongly affected by TiC content, but little influenced by extra Si and Al.

The effects of TiC, Si, and Al on the combustion front propagation velocity are presented in Figure 4, indicating a substantial decrease in the flame-front speed with TiC content from 6.7 mm/s for the elemental 3Ti-Si-2C sample to 2.3 mm/s for the TiC-containing sample of x = 0.7. As the layer-by-layer heat transfer from the reaction zone to an unburned region plays an important role in establishing combustion wave behavior, the propagation speed is strongly affected by the combustion front temperature. Such a great decrease in the combustion wave velocity could be a consequence of the dilution effect of TiC on combustion and the change in the reaction mechanism from a liquid–solid reaction mode to one dominated by solid-phase species, as discussed above. In particular, the combustion wave of the sample with x = 0.7 propagated in a spinning manner, which prolonged the total reaction time and reduced the propagation rate.

In addition, Figure 4 reveals that the addition of extra Si (y = 1.2) and Al (z = 0.1) had relatively minor effects on combustion velocity. The combustion velocity of the sample with extra Si of 20% was very close to that with stoichiometric Si. The addition of Al generally increased the combustion velocity by about 5%, possibly due to the formation of a liquid phase in the Ti-Si-C-Al system [32]. It should be noted that extra Si and Al also had little influence on the combustion temperature.

### 3.2. Composition and Microstructure Analyses of Synthesized Products

**Figure 5:**
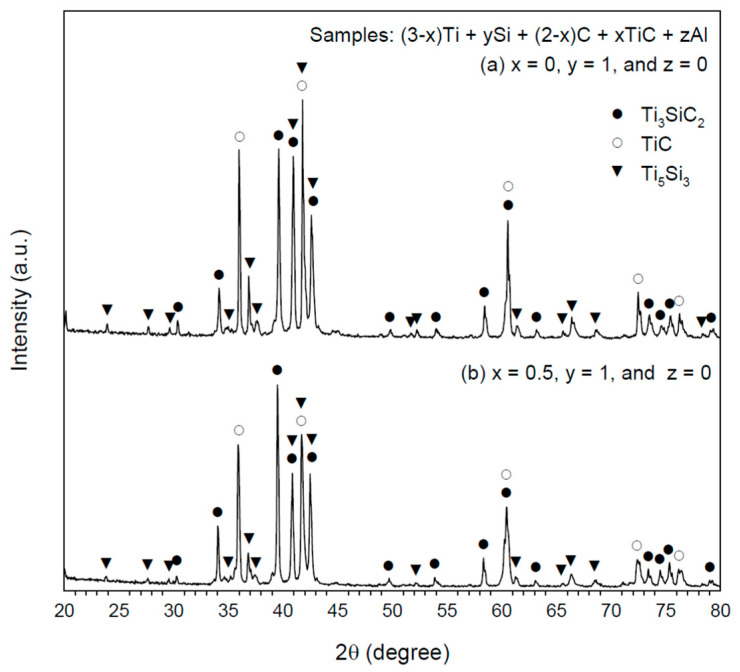
XRD patterns of the products synthesized from samples with composition parameters of (**a**) x = 0, y = 1, and z = 0 and (**b**) x = 0.5, y = 1, and z = 0.

Figure 5a,b show XRD patterns of the final products synthesized from the elemental 3Ti-Si-C sample and the TiC-containing sample of x = 0.5, respectively. No extra Si and Al were employed in the samples of Figure 5. The final products were composed of three constituent phases, Ti_3_SiC_2_, TiC, and Ti_5_Si_3_. The diffraction peak intensity of Ti_3_SiC_2_ relative to that of either TiC or Ti_5_Si_3_ is noticeably amplified in Figure 5b when compared with that of Figure 5a. This justified the role of TiC as the reactant in enhancing the formation of Ti_3_SiC_2_ by the SHS process because TiC is one of two major intermediates to form Ti_3_SiC_2_.

**Figure 6:**
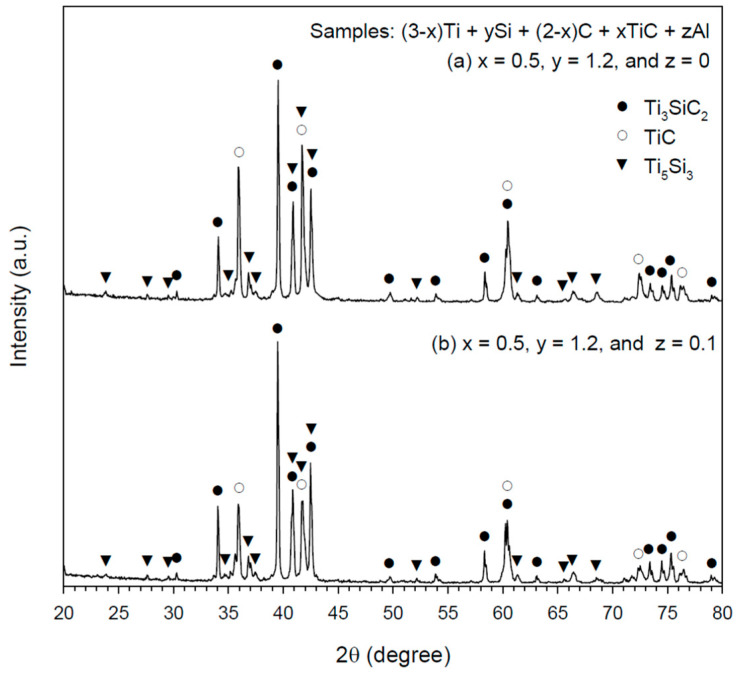
XRD patterns of the products synthesized from samples with composition parameters of (**a**) x = 0.5, y = 1.2, and z = 0 and (**b**) x = 0.5, y = 1.2, and z = 0.1.

The effects of Si and Al on the formation of Ti_3_SiC_2_ can be seen in Figure 6, which presents XRD spectra for TiC-containing samples of x = 0.5 with excess Si and the addition of Al. Figure 6a reveals that for the Si-rich sample without Al additions, the dominancy of Ti_3_SiC_2_ over TiC and Ti_5_Si_3_ was stronger than that observed in Figure 5b. This indicated that the sample with Si in excess of 20 mol.% improved the yield of Ti_3_SiC_2_ as extra Si compensated for the evaporation loss of Si at high temperatures. For the Si-rich/Al-added sample, Figure 6b indicates that the addition of a small amount of Al further augmented the XRD peak intensity of Ti_3_SiC_2_. According to Zhang et al. [32], Al can aid the formation of a liquid phase in the Ti-Si-C-Al system, and the liquid facilitates the diffusion of Ti and Si atoms and promotes the evolution of Ti_3_SiC_2_. As a reaction promoter, Sun et al. [33] pointed out that Al of a small quantity could evaporate from the grain boundary, rather than forming a solid solution in Ti_3_SiC_2_.

**Figure 7:**
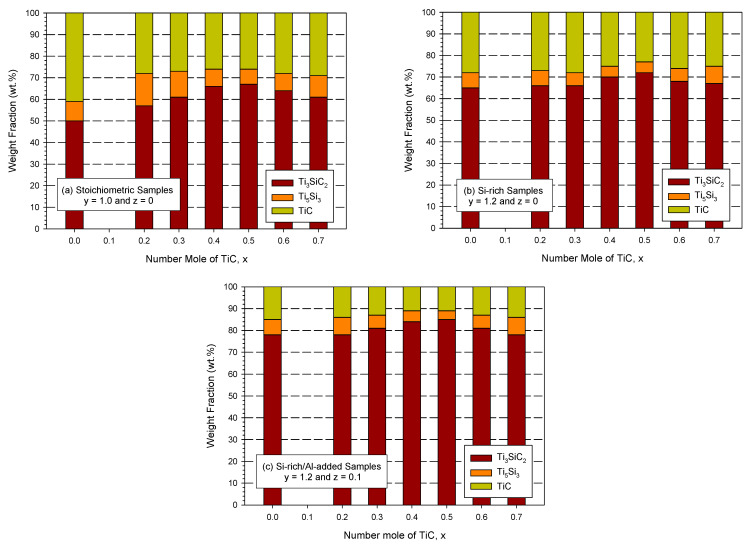
Weight fractions of Ti_3_SiC_2_, Ti_5_Si_3_, and TiC in the products synthesized from (**a**) stoichiometric samples of y = 1 and z = 0, (**b**) Si-rich samples of y = 1.2 and z = 0, and (**c**) Si-rich/Al-added samples of y = 1.2 and z = 0.1.

The weight percentages of Ti_3_SiC_2_, TiC, and Ti_5_Si_3_ in the products associated with the three initial sample compositions were calculated and are presented in Figure 7a–c. For the stoichiometric samples with Ti:Si:C = 3:1:2 (i.e., y = 1 and z = 0), Figure 7a shows that for the TiC-free sample, the resulting product was composed of 50 wt.% Ti_3_SiC_2_, 41 wt.% TiC, and 9 wt.% Ti_5_Si_3_. The TiC-containing sample was found to increase the formation of Ti_3_SiC_2_; a maximum yield of 67 wt.% was detected at x = 0.5, in which the fractions of the other two phases were 26 wt.% TiC and 7 wt.% Ti_5_Si_3_. However, a further increase in the TiC content to x = 0.6 and 0.7 decreased the yield percentage of Ti_3_SiC_2_, possibly due to a reduced reaction temperature.

For the Si-rich samples of y = 1.2 and z = 0, the variations in Ti_3_SiC_2_, TiC, and Ti_5_Si_3_ fractions in the final products with the number of moles of TiC in the reactants are presented in Figure 7b. The contribution of excess Si to the production of Ti_3_SiC_2_ was justified, and the fraction of Ti_3_SiC_2_ reached between 66 and 72 wt.% for the TiC-containing samples. In addition, the content of TiC was about 25–27 wt.% and that of Ti_5_Si_3_ was around 5–7 wt.% for the products of the Si-rich samples.

The formation of Ti_3_SiC_2_ was further improved by the Si-rich/Al-added samples (y = 1.2 and z = 0.1). As unveiled in Figure 7c, the yield fraction of Ti_3_SiC_2_ exceeded 80 wt.% for the samples initially containing TiC between x = 0.3 and 0.6. The highest yield of Ti_3_SiC_2_, reaching 85 wt.%, was obtained from the sample of x = 0.5 and the product also contained 11 wt.% TiC and 4 wt.% Ti_5_Si_3_. Even for the TiC-free sample, as shown in Figure 7c, the fraction of Ti_3_SiC_2_ increased up to 78 wt.%.

**Figure 8:**
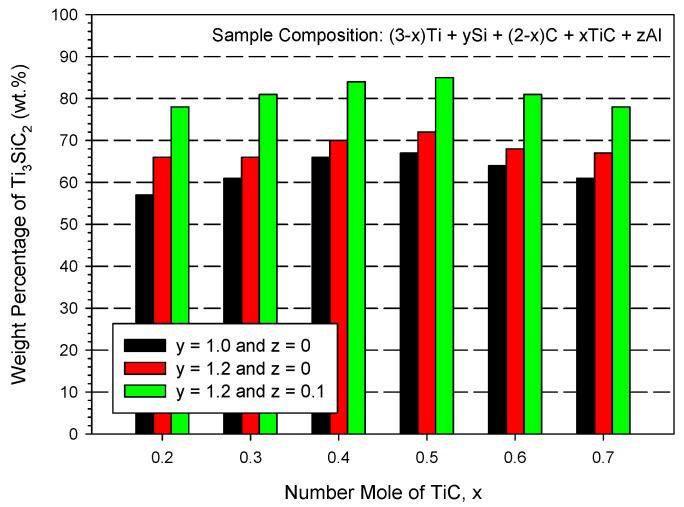
Variations in weight percentage of Ti_3_SiC_2_ formed in the final products with initial TiC content, excess Si, and Al additions.

Figure 8 makes a comparison of the yield fractions of Ti_3_SiC_2_ between the samples of three different compositions. It was evident that both extra Si and Al contributed to the formation of Ti_3_SiC_2_, and a larger increase in the yield percentage was achieved by the addition of Al. In this study, the highest yield of Ti_3_SiC_2_ was produced by the sample of 2.5Ti + 1.2Si + 1.5C + 0.5TiC + 0.1Al.

**Figure 9:**
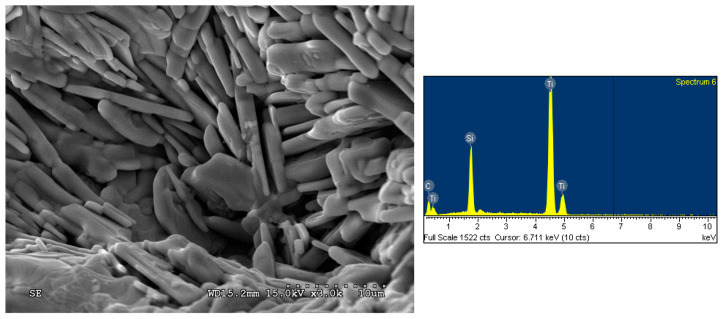
SEM image and EDS spectrum of the product synthesized from the Si-rich sample with composition parameters of x = 0.5, y = 1.2, and z = 0.

**Figure 10:**
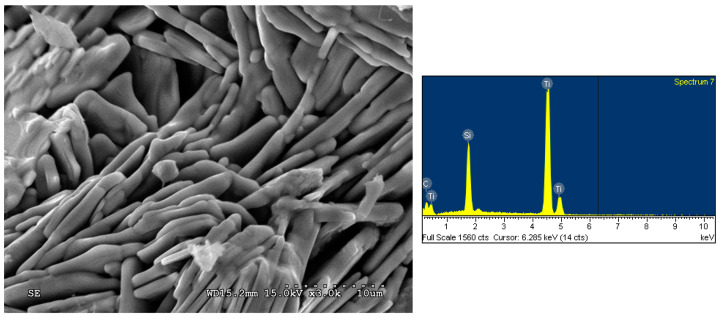
SEM image and EDS spectrum of the product synthesized from the Si-rich/Al-added sample with composition parameters of x = 0.5, y = 1.2, and z = 0.1.

The SEM image and EDS spectrum illustrated in Figure 9 display the fracture surface microstructure and atomic ratio of the product synthesized from the TiC-containing and Si-rich samples of x = 0.5, y = 1.2, and z = 0. Plate-like grains forming a layered microstructure, which is typical of the MAX ternary carbide, are clearly seen; the Ti_3_SiC_2_ platelets were 0.5–1.0 μm in thickness and about 10 μm in length. An atomic ratio of Ti:Si:C = 52.6:13.8:33.6 matching well with Ti_3_SiC_2_ was obtained from the EDS analysis. The microstructure and EDS element spectrum presented in Figure 10 were associated with the product of the Si-rich/Al-added samples of x = 0.5, y = 1.2, and z = 0.1. Similarly, plate-like Ti_3_SiC_2_ grains closely packed into a laminated configuration were observed. The atomic ratio of Ti:Si:C = 51.6:15.0:33.4 provided by EDS confirmed the formation of Ti_3_SiC_2_.

Table 1 summarizes the Ti_3_SiC_2_-related products fabricated by various methods with different starting reactant mixtures. For the formation of single-phase Ti_3_SiC_2_, SPS and reactive sintering are suitable methods. These two methods require high operating temperatures (about 1300–1500 °C) for 20–30 min or even 2–3 h. On the other hand, the SHS technique is an energy-efficient and time-saving fabrication route, and is suitable for the synthesis of Ti_3_SiC_2_/TiC/Ti_5_Si_3_ composites. As listed in Table 1, the addition of a small amount of Al has frequently been applied to improve the yield of Ti_3_SiC_2_ and slightly Si-rich mixtures have been adopted. Moreover, TiC and SiC have also been considered as the reactants in different fabrication methods.

## 4. Conclusions

The formation of Ti_3_SiC_2_ was investigated with the SHS method using TiC-containing reactant compacts with three compositions: an exact 312 stoichiometry of Ti:Si:C = 3:1:2, a composition with excess Si of 20 mol.% at Ti:Si:C = 3:1.2:2, and a Si-rich/Al-added composition of Ti:Si:C:Al = 3:1.2:2:0.1. The initial reactant composition could be expressed by (3 − x)Ti + ySi + (2 − x)C + xTiC + zAl, with x ranging from 0 to 0.7, y = 1.0 and 1.2, and z = 0 and 0.1.

Experimental evidence indicated that solid-state combustion was highly exothermic and a self-sustaining combustion synthesis process was readily achieved upon ignition. Combustion wave velocity and reaction temperatures substantially decreased from 6.7 to 2.3 mm/s and 1464 to 1200 °C, respectively, as the TiC content in the green samples increased from x = 0 to x = 0.7. This was mainly attributed to the dilution effect of TiC on combustion. The XRD analysis identified that final products were composed of Ti_3_SiC_2_ and two intermediate phases, TiC and Ti_5_Si_3_. When compared with TiC-free samples (x = 0), the TiC-containing samples benefited the formation of Ti_3_SiC_2_. The optimum content of TiC was found to be x = 0.5, beyond which the yield of Ti_3_SiC_2_ declined due to a decrease in combustion temperature. Even though extra Si (y = 1.2) and Al (z = 0.1) had almost no effect on combustion wave velocity and temperature, they enhanced the formation of Ti_3_SiC_2_ to a great extent. Considering the TiC-containing samples of x = 0.5, the weight fraction of Ti_3_SiC_2_ formed in the final products increased from 67 wt.% for the sample of Ti:Si:C = 3:1:2 to 72 wt.% for the Si-rich sample, and further to 85 wt.% for the Si-rich/Al-added sample. This justified the contribution of excess Si to subsidize for its loss and the role of Al as an effective reaction promoter. The as-synthesized Ti_3_SiC_2_ grains were in a plate-like shape with a thickness of 0.5–1.0 μm and length of about 10 μm. The Ti_3_SiC_2_ grains were closely stacked into a laminated configuration, which is typical of a MAX microstructure.

One of main limitations to further commercialize MAX phases is the development of a suitable synthesis process to produce large quantities of highly pure MAX powders at an affordable cost [11]. Synthesis of MAX powders by the SHS method was shown to successfully produce industrial quantities at reasonable prices. More efforts and attention are required to synthesize and scale-up MAX phase powders of sufficient purities or desired compositions. This study represents a substantial advance on the formation of Ti_3_SiC_2_/TiC/Ti_5_Si_3_ composites with controllable compositions by SHS.

## Figures and Tables

**Table 1:** Ti_3_SiC_2_ products fabricated by different methods with various starting reactants.

Methods	Reactant Mixtures	Synthesized Products	Refs.
SPS (1500 °C for 20 min)	3Ti/SiC/C	Ti_3_SiC_2_/TiC/Ti_5_Si_3_ composite	[20]
SPS (1400 °C for 15 min)	Ti/1.1Si/2TiC/0.2Al 2.2Si/3TiC/0.2Al	Pure Ti_3_SiC_2_ Ti_3_SiC_2_/SiC composite	[21]
SPS (1280 °C for 36 min)	3Ti/Si/2C/0.2Al	Pure Ti_3_SiC_2_	[32]
SPS (1200 °C for 30 min)	3Ti/SiC/C/0.15Al	Pure Ti_3_SiC_2_	[33]
Reactive sintering (1350 °C for 3 h)	3TiH_2_/1.2Si/2C	Pure Ti_3_SiC_2_	[31]
Reactive sintering (1280 °C for 1 h)	4Ti/TiC/2SiC/0.2Al	Ti_3_SiC_2_/TiC composite	[34]
Reactive sintering (1400 °C for 2 h)	3Ti/1.5Si/1.9C/0.5Al	Pure Ti_3_SiC_2_	[35]
Reactive sintering (1300 °C for 2 h)	Ti/Si/2TiC/0.2Al	Pure Ti_3_SiC_2_	[41]
SHS	3Ti/Si/2C, Ti/Si/2TiC, and 3Ti/SiC/C	Ti_3_SiC_2_/TiC/Ti_5_Si_3_ composite	[25]
SHS	3Ti/Si/2C/0.2Al	Ti_3_SiC_2_/TiC composite	[36]
SHS	2.5Ti/1.2Si/1.5C/0.5TiC/0.1Al	Ti_3_SiC_2_/TiC/Ti_5_Si_3_ composite	This work

## Data Availability

Data presented in this study are available in the article.
